# Sacrificial Core-Based Electrospinning: A Facile and Versatile Approach to Fabricate Devices for Potential Cell and Tissue Encapsulation Applications

**DOI:** 10.3390/nano8100863

**Published:** 2018-10-21

**Authors:** Naresh Kasoju, Julian George, Hua Ye, Zhanfeng Cui

**Affiliations:** 1 Institute of Biomedical Engineering, Department of Engineering Science, University of Oxford, Oxford OX3 7DQ, UK; naresh.kasoju@sctimst.ac.in (N.K.); julian.george@eng.ox.ac.uk (J.G.); 2 Current affiliation: Division of Tissue Culture, Department of Applied Biology, Biomedical Technology Wing, Sree Chitra Tirunal Institute for Medical Sciences and Technology, Thiruvananthapuram 695 012, India

**Keywords:** electrospinning, 3D printing, nanofibers, encapsulation, protein diffusion, *in vivo* tissue engineering, immuno-isolation, transplantation

## Abstract

Electrospinning uses an electric field to produce fine fibers of nano and micron scale diameters from polymer solutions. Despite innovation in jet initiation, jet path control and fiber collection, it is common to only fabricate planar and tubular-shaped electrospun products. For applications that encapsulate cells and tissues inside a porous container, it is useful to develop biocompatible hollow core-containing devices. To this end, by introducing a 3D-printed framework containing a sodium chloride pellet (sacrificial core) as the collector and through post-electrospinning dissolution of the sacrificial core, we demonstrate that hollow core containing polyamide 66 (nylon 66) devices can be easily fabricated for use as cell encapsulation systems. ATR-FTIR and TG/DTA studies were used to verify that the bulk properties of the electrospun device were not altered by contact with the salt pellet during fiber collection. Protein diffusion investigations demonstrated that the capsule allowed free diffusion of model biomolecules (insulin, albumin and Ig G). Cell encapsulation studies with model cell types (fibroblasts and lymphocytes) revealed that the capsule supports the viability of encapsulated cells inside the capsule whilst compartmentalizing immune cells outside of the capsule. Taken together, the use of a salt pellet as a sacrificial core within a 3D printed framework to support fiber collection, as well as the ability to easily remove this core using aqueous dissolution, results in a biocompatible device that can be tailored for use in cell and tissue encapsulation applications.

## 1. Introduction

Electrospinning is a process in which a charged polymer jet is deposited on a grounded collector. Introduced in the early 1900s, the technology is now a well-established and an intensively investigated way of producing continuous fibers from a wide variety of polymer solutions, with diameters ranging from several microns down to several nanometers [1]. With a high surface area to volume ratio, tunable porosity and flexibility in surface modification, electrospun fibers have been successfully used in various applications including the fabrication of protective clothing, electronics, catalysis and pharmaceutics, as well as in the fields of filtration, environmental engineering and regenerative medicine. Typically, a syringe is used to load the polymer solution and a syringe pump controls the solution flow rate from a tip charged by a high-voltage power unit. The charged polymer jet takes a torturous path, elongating and drying as it is pulled towards a grounded collector. Many modifications to the electrospinning setup have been investigated over the last couple of decades [2,3]. For instance, a co-axial electrospinning system was developed to fabricate core-shell fibrous structures, wherein the inclusion of a sacrificial core can be used to yield hollow fibers or the inclusion of multiple cores can be used to yield multi-core fibers [4,5]. An axillary cylindrical electrode has also been used to facilitate multiple spinneret electrospinning that dramatically increases the rate of fiber production [6]. Similarly, conductive patterned collector-based electrospinning has been used to generate fibrous materials with controlled architectures [7].

Despite innovations in jet facilitation and control over path and fiber collection, the most commonly fabricated electrospun forms are flat membranes produced using flat collector plates, and tubular structures produced using rod-like collectors. In the context of biomedical applications, flat membranes are widely used as substrates for culturing a variety of cells and engineering tissues, such as skin [8], whereas the tubular structures are used to engineer tissues such as blood vessels and neural conduits [9]. Beyond these two forms, there is demand and scope for novel products and devices such as encapsulation systems that expand the reach of electrospinning applications in regenerative medicine. For example, a common approach used to treat type 1 diabetes mellitus is to infuse pancreatic islets into the hepatic portal vein; however, this implantation site has proven to be sub-optimal. The development of devices that encapsulate cells within a protective environment would facilitate the choice of alternative sites. In the field of tissue engineering, current *in vitro* approaches are unable to replicate native cell niche environments and hence there is interest in the development of *in vivo* bio-incubators that better replicate the tissue environment. Similarly, in the field of allo- or xeno-cell and tissue transplantation, the side effects of using immune suppressants are undesirable and there is increasing demand to develop suitable immuno-isolating devices [10,11].

In this report, we describe the fabrication of a macro-scale encapsulation device, produced through manipulation of the collection of electrospun fibers. It was hypothesized that the introduction of a water-soluble pellet (sacrificial core), such as a custom-made sodium chloride pellet, as the collector would allow for post-electrospinning dissolution of this sacrificial core, making it possible to fabricate a hollow polymeric macro-scale encapsulation device. Fabrication of capsules with varying size, shape and thickness would be possible by modifying the size, shape and thickness of the sacrificial core. It was further hypothesized that a framework would be required to prevent the collapse of the electrospun device upon sacrificial core dissolution, and therefore, it was proposed that a 3D printed framework would be used to support the sacrificial core during preparation and electrospinning, and that this framework would remain in place to prevent collapse following core dissolution. Polyamide 6,6 (PA66) was selected as the polymer of choice. This is a non-degradable and biocompatible polymer that has been widely explored in biomedical applications. The electrospun capsules produced were evaluated through the use of scanning electron microscopy (SEM), attenuated total reflectance Fourier transform infrared spectroscopy (ATR-FTIR) and simultaneous thermo gravimetric/differential thermal analysis (TG/DTA). Finally, protein diffusion studies using model biomolecules (insulin, albumin and Ig G) were performed to study the free diffusion of essential proteins through the capsule walls, and cell encapsulation studies with model cell types (fibroblasts and lymphocytes) were performed to investigate whether the device could support the viability of encapsulated cells whilst restricting immune cell access from the surrounding space.

## 2. Materials and Methods

### 2.1. Materials

Polyamide 6,6 (PA66, also known as nylon 6,6 or Poly(N,N′-hexamethyleneadipinediamide)), 2,2,2-Trifluoroethanol (TFE), formic acid (FA), 1,1,1,3,3,3-Hexafluoro-2-propanol (HFIP), human insulin, human Ig G and bovine serum albumin (BSA) were purchased from Sigma-Aldrich, Dorset, UK, and the protein determination kit was purchased from Cayman Chemical, Ann Arbor, MI, USA. Dulbecco’s Modified Eagle Medium (DMEM), Roswell Park Memorial Institute 1640 (RPMI-1640) medium, fetal bovine serum (FBS), trypsin-Ethylenediaminetetraacetic acid (EDTA), phosphate-buffered saline (PBS, 1×), Penicillin-Streptomycin (Pen-Strep, 100×) and Alamar blue were purchased from Thermo Fisher Scientific, Waltham, MA, USA.

### 2.2. Preparation of Macroscale Capsules by Sacrificial Core Electrospinning

#### 2.2.1. Preparation of a 3D Printed Supporting Framework

**Figure 1:**
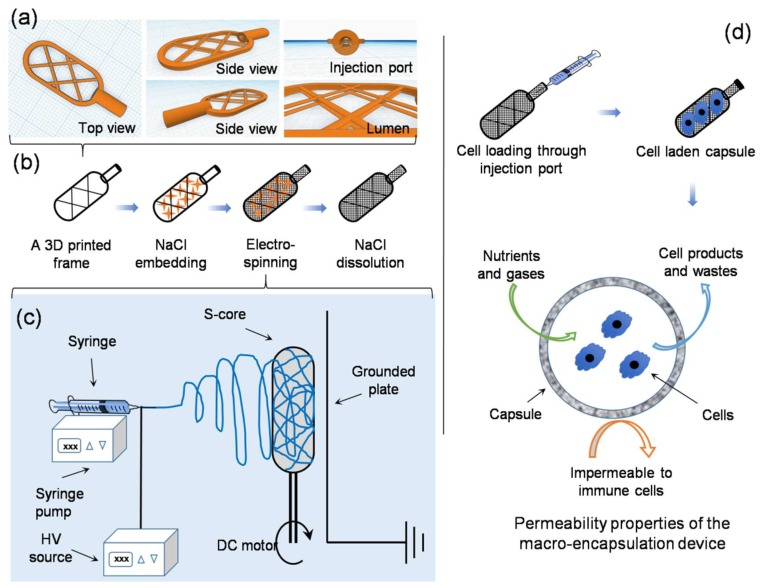
Schematic showing sacrificial core electrospinning capsule fabrication and use: (**a**) Images show the CAD 3D model of the supporting framework (frame). (**b**) Work flow showing the steps involved in device assembly, detailing supporting framework production, salt embedding, electrospinning and salt dissolution. (**c**) Electrospinning setup, highlighting how the salt aggregate contacting supporting frame was placed directly in front of the grounded collecting plate and rotated during fiber collection. (**d**) Loading of the electrospun capsule with cells for use in cell and tissue encapsulation applications, highlighting how the capsule allows free diffusion on nutrients, gases, cellular products and waste, whilst blocking access to immune cell penetration.

An online computer-assisted design tool (Tinkercad, AUTODESK, San Rafael, CA, USA) was used to prepare a 3D stereolithographic design in STL (stereolithography) file format. As shown in Figure 1a, a typical framework contains an external thick skeletal structure with a thin internal lattice of crisscross elements used to create a luminal space. An opening injection port to one side allows access to the internal space. The design was uploaded to a computer-assisted 3D printer manufacturing system (Form 2, FormLabs, Somerville, MA, USA). The 3D printer uses a laser to cure solid isotropic parts from a liquid photopolymer resin. A biocompatible resin was used (EN-ISO 10993-1:2009/AC:2010, USP Class VI, Dental SG, FormLabs, Somerville, MA, USA). Removal of non-cured resin was achieved by post-fabrication washing in absolute ethanol for 15 min, as per the supplier’s recommendation.

#### 2.2.2. Embedding Salt into the Printed Framework

The printed framework was kept on a flat surface and sodium chloride crystals were packed into the lumen of the framework. Water was used to wet the packed crystals, partially solubilizing the salt at the crystal boundaries, and fusing the crystals into a single aggregate. After allowing to dry at room temperature on an open bench for 24 h, the salt pellet containing the framework was ready to be used as a collector during electrospinning (Figure 1b).

#### 2.2.3. Sacrificial Core Electrospinning

PA66 pellets were suspended in different organic solvents at various concentrations (see Table 1) and stirred continuously until completely dissolved. The solution was electrospun at ambient temperature (20–22 °C) in a low humidity environment (25 ± 5% RH – relative humidity, controlled by purging the chamber with nitrogen gas). The sacrificial core electrospinning setup included a high-voltage power supply, a digital syringe pump and a specialized collecting unit, composed of the sacrificial core and printed framework connected to a DC (direct current) motor and placed directly in front of a grounded metal plate (Figure 1c). The entire setup was enclosed inside a custom-made plastic chamber with fume extraction and safety interlocks. To reduce variability in fiber diameter and packing density, the DC motor was run at a constant speed during the fabrication of all capsules. Systematic optimization was performed by varying the processing parameters, as detailed in Table 1.

### 2.3. Characterization Studies

The morphological features of the electrospun PA66 materials were imaged and analyzed using SEM (Evo LS15, Carl Zeiss, Oberkochen, Germany). For this purpose, the samples were air dried in a chemical fume hood. They were then mounted on the specimen holder with double adhesive electro-conductive carbon tape, sputter-coated with platinum for 90 seconds under argon gas in a coating unit (Polaron range SC7620, Quorum Technologies Ltd, East Sussex, UK), and imaged. Fiber diameter was calculated manually by analyzing the images using Image J software (NIH, Bethesda, MD, USA).

Changes to the chemistry of PA66 after electrospinning were tracked using Attenuated Total Reflection-Fourier-transform infrared spectroscopy (ATR-FTIR, Tensor 37, Bruker, Billerica, MA, USA). For each measurement, 16 scans were recorded with a resolution of 1 cm^−1^ and wave numbers ranging from 800 to 4000 cm^−1^. During the measurements, the instrument was continuously purged with nitrogen using blow-off from a liquid nitrogen tank to eliminate the spectral contributions of atmospheric water vapor.

The thermal properties of PA66 before and after electrospinning were analyzed by simultaneous thermogravimetry/differential thermal analysis (TG/DTA, Diamond, PerkinElmer, Waltham, MA, USA). In each measurement, the sample was heated from 40 to 600 °C at a rate of 10 °C/min. During the measurements, the instrument was continuously purged with nitrogen gas at a rate of 20 mL/min to eliminate the spectral alterations due to the presence of ambient air.

### 2.4. Protein Diffusion Assay

PA66 capsules (1 cm width × 2 cm length × 1 mm thickness) made from 7.5% (*w*/*v*) polymer solution with 15 kV voltage, 15 cm distance and 0.5 mL/min flow rate, were sterilized by autoclaving and saturated with 1× PBS for 3 h in an incubator (5% CO_2_, 37 °C). Capsules were placed in a 6-well plate and simultaneously loaded with insulin, Ig G or BSA solution (1 mg/mL). The capsule loading port was tied tightly with a sterile nylon thread and kept in a CO_2_ incubator overnight. The contents were manually mixed at regular intervals. The spent protein solution released from the capsule was collected and assayed by the Bradford method following the kit instructions (Cayman Chemical, Ann Arbor, MI, USA). Briefly, 100 µL of diluted protein solution was mixed with equal amounts of assay reagent. The contents were incubated at room temperature for 5 min and the absorbance was read at 595 nm in a multi-well plate reader (Spectra Max i3x, Molecular Devices, San Jose, CA, USA). Results were compared with a standard curve made using known concentrations of insulin, Ig G and BSA. 

### 2.5. Cell Encapsulation Studies

Human dermal fibroblasts (HDF, Thermo Fisher Scientific, Waltham, MA, USA) and Jurkat cells (ATCC, Manassas, VA, USA) were routinely cultured in DMEM and RMPI-1640 medium respectively, in a CO_2_ incubator. The medium was supplemented with 10% FBS and 1% Pen-Strep. PA66 capsules were prepared as described in Section 2.4. The capsules were saturated in complete medium overnight, and 5.0 × 10^5^ cells/mL of exponentially growing cells were loaded into each capsule through the injection port. The injection port was closed tightly with a sterile nylon thread, and they were cultured in a CO_2_ incubator. After 3 d of incubation, the cell-laden capsules were transferred to a fresh plate with fresh medium. The presence of cells, both inside and outside the capsules, was measured using the metabolic Alamar blue assay, comparing the medium in the spent and receiving plates. In the case of HDF cells, if any cells migrate out of the capsule, they would attach to the bottom of the plate; the spent medium was exchanged directly with the fresh medium. In the case of Jurkat cells, the spent medium was centrifuged to collect cells at the bottom of the well prior to replacing the spent medium with fresh medium. Briefly, 1 mL of fresh medium together with 100µL of Alamar blue reagent was added to both the wells containing the cell-laden capsules and the wells of the spent plate. The contents were mixed well and incubated in a CO_2_ incubator. After 4 h of incubation, the absorbance was measured at a wavelength of 570 nm, using a 600 nm wavelength as a reference. An equal number of cells cultured in a standard tissue culture treated polystyrene (TCPS) plate, under similar conditions, were treated as the positive control. Cell-free Alamar blue reagent, under similar conditions, was treated as the negative control. The cell viability was measured using the following Equation:

(1) $$\;\mathrm{Cell}\;\mathrm{viability}\%=\frac{\mathrm{Absorbance}\;\mathrm{of}\;\mathrm{Test}\;\mathrm{Sample}}{\mathrm{Absorbance}\;\mathrm{of}\;\mathrm{Control}\;\mathrm{Sample}}\times 100\;$$

### 2.6. Statistical Analysis

The quantitative values were averaged and expressed as mean ± standard deviation. One-way ANOVA was performed to compare significance across groups, and the differences were denoted with *, # and ‡ representing *p* < 0.05, *p* < 0.01 and *p* < 0.001 respectively.

## 3. Results and Discussion

**Figure 2:**
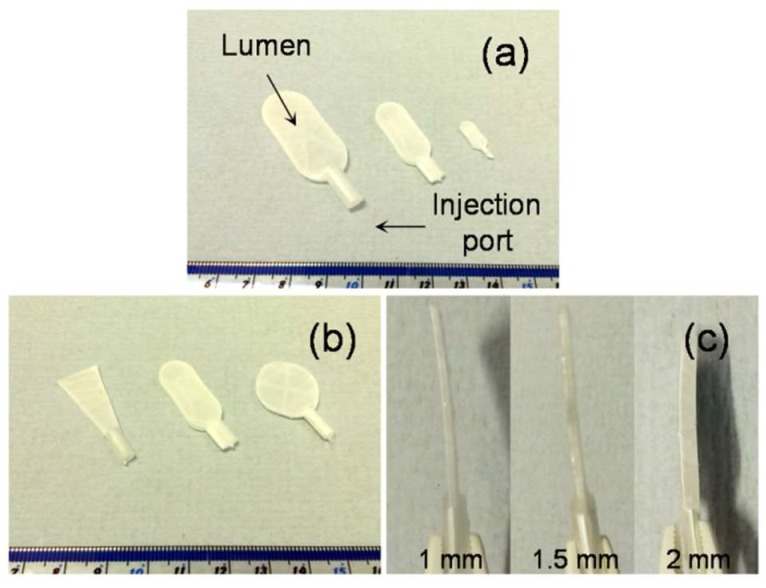
Sacrificial core electrospun products: With sacrificial core-based electrospinning, it was possible to prepare PA66 macro-capsules with variable size (**a**), shape (**b**), and thickness (**c**).

Inspired by techniques such as freeze-drying [12] and salt-leaching [13] where ice crystals or salt crystals are used as porogens, it was hypothesized that it should be possible to use a similar approach to modify the macroscale form of fibers collected using conventional electrospinning. This setup, termed “sacrificial core electrospinning”, could be used to produce devices for potential cell and tissue encapsulation applications. As presented in Figure 1, the setup was simple and extends conventional systems though inclusion of a sacrificial core (a sodium chloride pellet) rotated directly in front of a grounded plate. Fibers deposited onto the pellet conform to the shape and size of the pellet. After electrospinning, the sacrificial core pellet can be dissolved by suspending in ultrapure water. Whilst this results in the creation of a hollow capsule with a port for cell or tissue injection, the electrospun fibers do not provide sufficient internal support and the device is susceptible to collapse (Figure S1). To overcome this issue, a custom-made 3D printed supporting frame was developed (Figure S2) and used both as a former for the sacrificial core and to provide internal support once the core has been dissolved (it is worthwhile to mention here that use of 3D printed frame without salt embedding was not efficient - check Figure S3). Using this approach, it was possible to successfully fabricate devices with varying length, width (Figure 2a), shape (Figure 2b) and thickness (Figure 2c).

**Figure 3:**
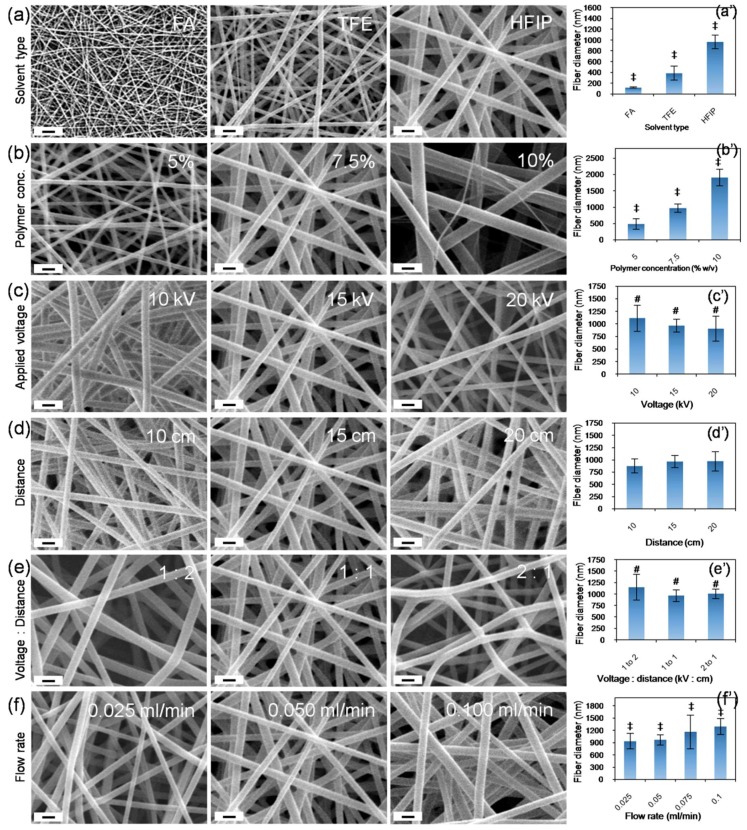
Effect of electrospinning parameters on fiber morphology: scanning electron microscopy (SEM) images of electrospun PA66 samples showing the effects of solvent type (**a**), polymer concentration (**b**), voltage (**c**), distance (**d**), voltage–to–distance ratio (**e**), and flow rate (**f**). Corresponding plots of fiber diameter are presented in (**a’**) to (**f’**) respectively. Fiber width was analyzed by manually measuring the width of individual fibers against a given scale using Image J software (n = 25). # and ‡ denotes statistical differences at *p* < 0.01 and *p* < 0.001 respectively. Scale bar = 2 µm.

Whilst conventional electrospinning techniques enable control to be gained over the microscale properties of fiber diameter and density, the use of a printed framework and salt core facilities the ability to control the macro-scale features of the fabricated construct. Of the 21 parameters commonly observed to influence fiber formation and the properties of the resultant product, we studied the effects of solvent type, polymer concentration, flow rate, applied voltage, tip-to-collector distance and voltage–to–distance ratio using polyamide 66 (PA66 or nylon 66) (Table 1 and Figure 3) [14]. In accordance with the literature, electrospunPA66 dissolved in formic acid (FA) resulted in thinner fibers (121 ± 12 nm), in comparison to PA66 electrospun in 2,2,2-Trifluoroethanol (TFE, 388 ± 127 nm) or 1,1,1,3,3,3-Hexafluoro-2-propanol (HFIP, 967 ± 127 nm) (Figure 3a). Differences in the evaporation rate of the solvents, as well as the conductivity and viscosity of the resultant solutions may have also contributed to the differences observed in fiber diameter [14]. Similarly, an increase in the PA66 concentration from 5 to 7.5 to 10% (*w*/*v*) resulted in an increase in fiber diameter from 486 ± 159 nm to 967 ± 127 nm to 1908 ± 258 nm respectively (Figure 3b). The viscosity and elastic modulus of the solution increases with polymer concentration and this may be attributable for the changes in the fiber diameter observed [14]. The change in fiber diameter in relation to voltage was found to follow a slight inverse relationship, with fiber diameters of 1113 ± 258 nm, 967 ± 127 nm and 904 ± 247 nm recorded at 10, 15 and 20 kV (Figure 3c). These changes may be attributed to the differences in volumetric charge density associated with the applied voltage, which would significantly affect the initial jet diameter at the tip of the Taylor cone [14]. Whilst the distance (Figure 3d) and the voltage–to–distance ratio (Figure 3e) do not seem to have any significant influence on fiber diameter, we noticed a change in fiber morphology in relation to the voltage–to–distance ratio. Finally, the fiber diameters were found to be 937 ± 189 nm, 967 ± 127 nm, 1165 ± 412 nm and 1292 ± 194 nm, when the flow rates were 0.025, 0.050, 0.075 and 0.100 mL/min respectively (Figure 3f). It has previously been noted that, with other parameters constant, the lowest flow rate typically results in a small jet radius that yields the thinnest fibers, whereas higher flow rates typically result in a larger jet radius and yield thicker fibers [14]. Our observations were in agreement with earlier reports [14,15,16].

**Figure 4:**
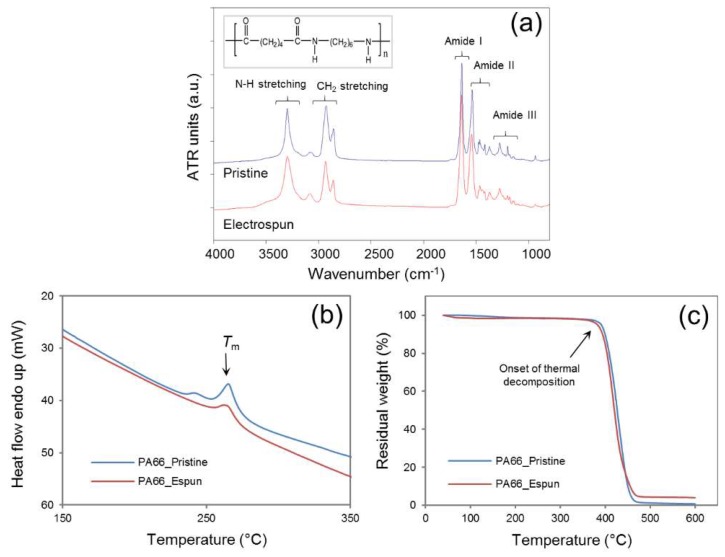
Material characteristics before and after sacrificial core electrospinning: Attenuated Total Reflection-Fourier-transform infrared spectroscopy (ATR-FTIR) (**a**), and thermogravimetry /differential thermal analysis (TG/DTA) (**b**—DSC, **c**—TGA) confirmed that the use of sodium chloride within the collector has no significant influence on the bulk properties of the polymer (PA66).

The bulk properties of the polymer were compared before and after the electrospinning process. Electricity, a physical property, does not typically affect the material properties of the polymer unless reactive additives are present in the polymer solution. In the current approach, the sodium chloride core was in direct contact with the deposited fibers within the electric field. Attenuated total reflectance—Fourier transform infrared spectroscopy (ATR-FTIR) revealed similar spectral patterns with characteristic peaks related to N–H stretching at ~3330 cm^−1^, CH_2_ stretching at ~2840 cm^−1^, amide I at ~1650 cm^−1^, amide II at ~1545 cm^−1^, and amide III at ~1370 cm^−1^ [15,17] (Figure 4a). A simultaneous TG/DTA investigation also suggested comparable heat flow patterns with a characteristic peak melting temperature (*T*_m_) at ~264.5 °C for pristine and ~262.5 °C for electrospun PA66, and decomposition patterns with the onset decomposition temperature of ~380 °C for pristine and ~373 °C for electrospun PA66 samples (Figure 4b,c). The spectral peak values were in good agreement with previously reported values for PA66 [16,18]. Collectively, ATR-FTIR and TG/DTA studies confirmed that the sacrificial core electrospinning was similar to the typical electrospinning process and did not influence the bulk properties of the polymer used in this study.

**Figure 5:**
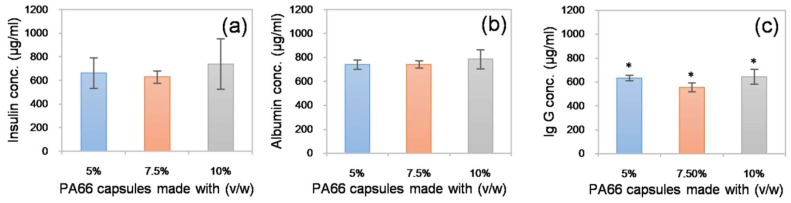
Protein diffusion study: Biomolecules such as insulin (**a**), albumin (**b**), and Ig G (**c**) loaded (1 mg/mL) into the sacrificial core electrospun capsules, were found to diffuse through the membrane into the incubation buffer. * denotes statistical differences at *p* < 0.05.

Since the devices fabricated using the sacrificial core electrospinning technique are intended to be used in cell and tissue encapsulation applications, it is of prime importance to verify the free movement of biomolecules, such as cytokines and growth factors, across the capsule walls. To investigate this, fabricated capsules were independently loaded with three model proteins: insulin, albumin and immunoglobulin G (Ig G) (1 mg/mL in PBS), with molecular weights of 5.8, 66 and 150 kDa, respectively, and incubated in protein-free PBS. Following overnight incubation at 37 °C, the capsule was removed, and the solution taken from around the capsule was assayed using the Bradford method. The presence of protein was detected in the solutions from all tested capsules (see Figure 5). However, minor differences were observed between the solutions from the 5, 7.5 and 10% (*w*/*v*) PA66 capsules. This may be due to the differences in the total surface area of the fibrous constructs. However, these differences were negligible. The results suggested that, in contrast to conventional hydrogel-based encapsulation systems, where the rate of biomolecule diffusion is restricted, the electrospun membranes produced in this study allow free diffusion of biomolecules. This may be beneficial for cell and tissues encapsulation applications that require rapid biomolecule transport [19].

**Figure 6:**
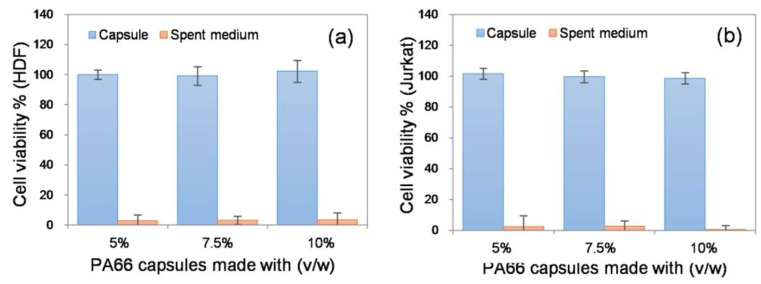
Cell encapsulation study: (**a**) Adherent cells (human dermis fibroblasts, HDF) and (**b**) non-adherent cells (lymphocytes from human peripheral blood, Jurkat cells) were encapsulated in the electrospun capsules. The number of cells present at day 3 remained unaffected in comparison to cultures on standard tissue culture-treated polystyrene dishes. Spent media showed no detectable cell activity, suggesting that the capsules effectively compartmentalized the cells. No statistical differences were found within the capsule and spent medium groups.

Finally, the ability of the electrospun capsule to support cells was assessed *in vitro* using two different model cell types: adherent fibroblasts (from human dermis) and non-adherent lymphocytes (Jurkat cells from human peripheral blood). In each case, 5.0 × 10^5^ cells were loaded into the capsule and the cell-laden capsule was incubated in a CO_2_ incubator. After 3 d of incubation, cell viability was indirectly measured using the metabolic Alamar blue assay. Cells cultured in a well plate under similar conditions were treated as the positive control. The purpose of the study was to investigate whether the porous structure of the capsule posed a barrier to diffusion that would affect the cell viability. Cell viability remained unchanged across all capsules tested (Figure 6). These results suggest that the electrospun membranes posed no limitation to the exchange of gases, nutrients and metabolic wastes essential for cell survival. We also collected the spent medium and performed further Alamar blue assays to determine if the encapsulated cells were released through the porous wall of the capsule. Both the fibroblasts and lymphocytes were not detected in the spent media, indicating that the porosity of the device (Figure S4) was efficient enough to restrict access to cells (Figure 6). The results indicate that the resultant electrospun device could be potentially used in various cell and tissue encapsulation applications. Considering the lymphocyte-based results, the capsule could also be used as an immuno-isolating system; however, detailed experiments are required to prove the immuno-protective properties of the device.

Previously, Lathuilièreet al., generated flat sheet devices consisting of an outer polymeric frame, porous membranes and a reinforcement mesh assembled using ultrasonic welding [20]. These devices were used for the implantation of genetically engineered allogeneic cells and passive immunization against amyloid-β. Similarly, Nyitrayet al., reported a two-step heat-sealing process to fabricate a polycaprolactone-based thin-film device and used this device to transplant pancreatic islets [21], whilst Park et al., described the assembly of a bacterial cellulose, collagen and alginate-based composite device for encapsulation of neuronal cells [22]. Recently, David et al., showed how a Polytetrafluoroethylene-based bilaminar device (Theracyte) can be used in the encapsulation of ovarian allograft to restore ovarian endocrine function [23]. While these reports suggest that there are a wide range of potential applications for bio-encapsulation technology, they also reveal the unmet need for an efficient macro-encapsulation device and illustrate the complexity of fabrication processes currently being investigated. In contrast, the sacrificial core electrospinning approach presented in this report is relatively simple as it follows well-known electrospinning principles and there is no complicated assembly or other processing steps involved. It is also versatile as it allows the fabrication of a device with a wide range of macro- and micro-scale features. The resultant device can be used as a cell-based drug delivery device or as a cell reservoir to treat conditions such as diabetes, neurological disorders and sensory diseases; however, the true efficiency of the device can only be determined by further *in vivo* studies.

## 4. Conclusions

In conclusion, this report describes a technological advancement in the field of electrospinning. While retaining all the benefits of the electrospinning process, a sodium chloride-based pellet (sacrificial core) has been introduced to act as a rotating collector, placed directly in front of a grounded plate. Subsequently, the dissolution of the salt pellet results in a hollow core containing capsule. With this technique, we have developed nylon (PA66)-based devices with varying macroscale features such as size (2 × 4, 1 × 2 and 0.5 × 1 cm), shape (rectangular, triangular and circular) and thickness (1, 1.5 and 2 mm). Additionally, it was possible to control the morphological properties of the capsule, such as fiber diameter by altering the conventional electrospinning parameters, including solvent type, polymer concentration, voltage, distance, voltage–to–distance ratio and flow rate. The results from the ATR-FTIR and TG/DTA studies performed suggest that the use of the sodium chloride pellet as the sacrificial core had no influence on the bulk properties of the polymer. Protein diffusion studies revealed that the capsules allowed free diffusion of biomolecules such as insulin, albumin and Ig G. Cell encapsulation studies with HDF and Jurkat cells revealed that the optimized capsules could be used to support the viability of encapsulated cells, whilst the walls of the capsules would restrict the ingress of immune cells. Further studies to unravel the potential of this system in various cell and tissue encapsulation applications are in progress.

## Figures and Tables

**Table 1:** Details of electrospinning parameters varied during optimization.

Parameter	Test Conditions	Constant Variables
Solvent type	FA, TFE and HFIP	Polymer conc. = 7.5% (*w*/*v*); Voltage = 15 kV; Distance = 15 cm; Flow rate = 0.05 mL/min
Polymer concentration	5, 7.5 and 10% (*w*/*v*)	Solvent = HFIP; Voltage = 15 kV; Distance = 15 cm; Flow rate = 0.05 mL/min
Voltage	10, 15, 20 and 30 kV	Polymer conc. = 7.5% (*w*/*v*); Solvent = HFIP; Distance = 15 cm; Flow rate = 0.05 mL/min
Tip to collector distance	10, 15, 20 and 30 cm	Polymer conc. = 7.5% (*w*/*v*); Solvent = HFIP; Voltage = 15 kV; Flow rate = 0.05 mL/min
Voltage: Tip to collector distance	1:2 (15 kV:30 cm), 1:1 (15 kV:15 cm) and 2:1 (30 kV:15 cm)	Polymer conc. = 7.5% (*w*/*v*); Solvent = HFIP; Flow rate = 0.05 mL/min
Flow rate	0.025, 0.050, 0.075 and 0.100 mL/min	Polymer conc. = 7.5% (*w*/*v*); Solvent = HFIP; Voltage = 15 kV; Distance = 15 cm

## Supplementary Materials

The following are available online at http://www.mdpi.com/2079-4991/8/10/863/s1, Figure S1: Physical state of the sacrificial core electrospun device without a supporting framework; Figure S2: A representative image of a 3D-printed supporting framework; Figure S3: Electrospinning onto 3D printed framework without inclusion of a NaCl pellet; Figure S4: Pore area calculations.
