# Supplementary material for: Sacrificial Core-Based Electrospinning: A Facile and Versatile Approach to Fabricate Devices for Potential Cell and Tissue Encapsulation Applications
Source: Nanomaterials (Basel). 2018 Oct 21;8(10):863. doi: 10.3390/nano8100863 (PMC6215104; doi:10.3390/nano8100863)
Supplement: Supplementary file 1 [file nanomaterials-08-00863-s001.pdf]

Supplementary

Sacrificial Core-Based Electrospinning: A Facile and Versatile Approach to Fabricate Devices for Potential Cell and Tissue Encapsulation Applications

Naresh Kasoju [1,2], Julian George[1], Hua Ye[1,*] and Zhanfeng Cui[1,*]

1 Institute of Biomedical Engineering, Department of Engineering Science, University of Oxford, Oxford OX3 7DQ, United Kingdom; naresh.kasoju@sctimst.ac.in (N.K.); julian.george@eng.ox.ac.uk (J.G.)
2 Current affiliation: Division of Tissue Culture, Department of Applied Biology, Biomedical Technology Wing, Sree Chitra Tirunal Institute for Medical Sciences and Technology, Thiruvananthapuram 695 012, India
* Correspondence: hua.ye@eng.ox.ac.uk (H.Y.); zhanfeng.cui@eng.ox.ac.uk (Z.C.)

Electrospinning onto the NaCl pellet without the 3D printed framework: NaCl can be cast into a given shape without the use of a printed framework through use of a shaped mold. Whilst electrospinning onto a rotating NaCl pellet is possible, the dissolution of the salt from the formed structure results in structure collapsed on removal from the dissolution bath (Figure S1). A 3D printed framework was used to ensure that the shape of the internal physical void was maintained (Figure S2).

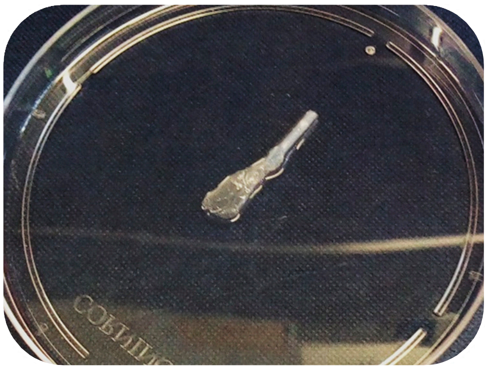

Figure S1. Physical state of the sacrificial core electrospun device without a supporting framework. It was found that the device collapses after the dissolution of the sacrificial core in ultrapure water. This was thought to be because of water surface tension drawing the fibers together on removal from the dissolution bath together with the lack of any supporting structure to prevent this.

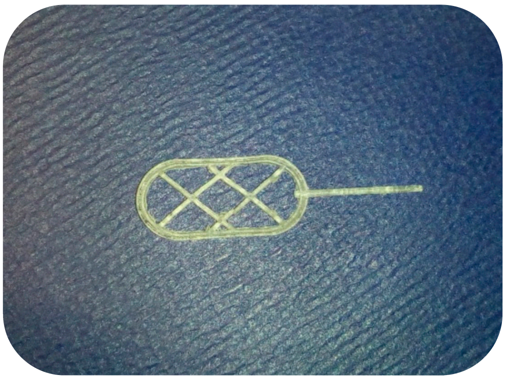

Figure S2. A representative image of a 3D-printed supporting framework: The framework supports can be used to template the shape of the NaCl pellet. Also, once the core has been dissolved in ultrapure water, the supporting framework is required to maintain the capsule shape.

Electrospinning onto the 3D the printed framework without inclusion of a NaCl pellet: We have investigated the use of printed frameworks with a thin structures (as presented in the main manuscript) and flexible structures (Figure S3, black color inherent to the resin used, Cat. No. RS-F2-FLGR-02, FormLabs). It was found that it was not possible to reliably fabricate a capsule without the inclusion of the NaCl pellet sacrificial core. Without the solid core it was found that the electrospun fibers not only filled the framework but also constricted the fine filaments of the

framework, causing it to collapse. However, when the chamber inside the mesh of the framework was filled with NaCl, the framework structure maintained it shape (Figure S3).

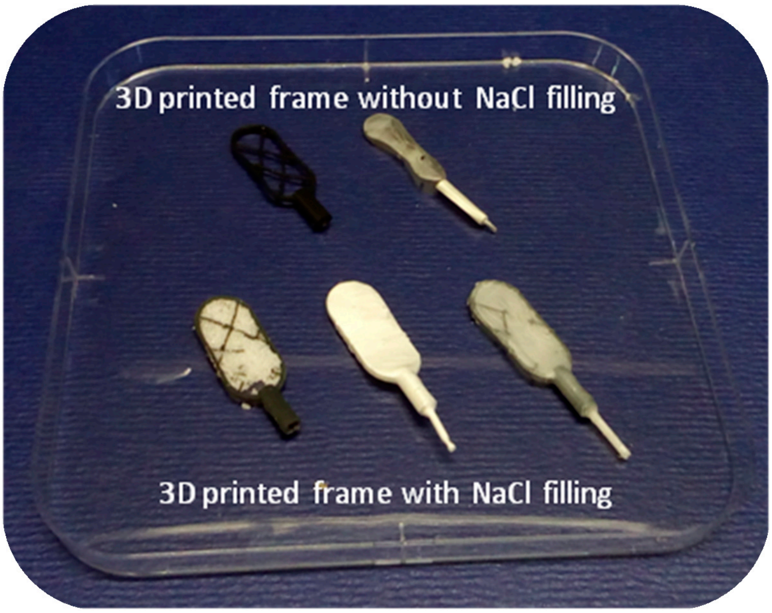

Figure S3. Electrospinning onto3D printed framework without inclusion of a NaCl pellet: A device made with a 3D printed framework without a NaCl solid core resulted in fibers entering the mesh work structure and the framework was often found to collapse due to the collector's rotation and fiber tensioning. However, a device made with NaCl solid core inside the framework retained its physical structure.

Pore size calculations: Pore size of the capsule fabricated at optimized parameters (i.e., 7.5% PA66 in HFIP, 15 kv voltage, 15 cm distance, 0.05 ml/min flow rate) was determined from corresponding SEM images following DiamaterJ plug-in in Image J software [1]. A total of 9 best segmented images were analyzed to determine the pore properties.

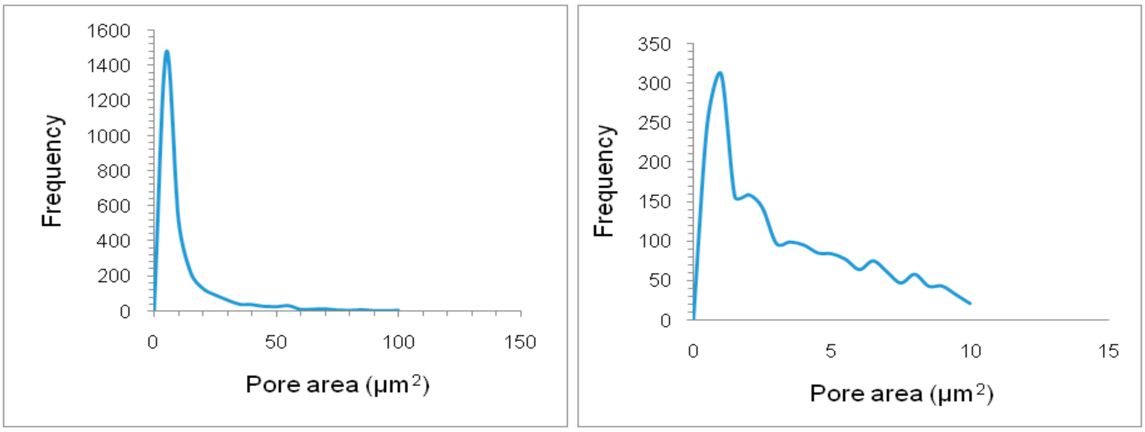

Figure S4. Pore area calculations: frequency distribution analysis (left - overall and right - zoomed) suggested that the majority of the pore areas were <10 um^2.

Cell encapsulation carrier: In the current study, cells were loaded into the core of the capsule in the form of suspension. Jurkat cells are anchorage independent cells and therefore would not adhere to the structure. HDF cells are anchorage dependent cells, and it was anticipated that the cells would adhere to the inside wall of the capsule. However, in clinical applications, a suitable carrier matrix such as non-degradable granular hydrogel could also be included to support the viability of

anchorage dependent cells. Such a matrix would act as both a cell carrier and as an additional protective layer that restricts immune access to the encapsulated cells.

Sacrificial core choice: To make electrospun capsules using the sacrificial core technique, we have previously fabricated pellets from sucrose crystals and gelatin sheets. The choice of using NaCl in the current study was based on the premise that NaCl was readily available, easy to remove, and was unlikely to interfere with downstream cell culture analysis. NaCl is also insoluble in the organic solvents under study (FA, HFIP and TFE). When electrospinning with an aqueous polymer in solution such as silk fibroin, the sacrificial pellet should be made from a water-insoluble material such as an organic polymer; once electrospinning is complete, it should be possible to dissolve the water-insoluble sacrificial core in a suitable organic solvent that is also poor solvent for silk fibroin.

Polymer choice: The intended application of the capsule discussed in the main article was as an implantable device for the transplantation and encapsulation of pancreatic islets. The materials chosen were needed to be both non-degradable and biocompatible, with PA66 meeting both these criteria.

References:

1. Nathan A. Hotaling, Kapil Bharti, Haydn Kriel, Carl G. Simon, DiameterJ: A validated open source nanofiber diameter measurement tool. Biomaterials 61: 327–338, 2015.
